# Molecular Biomarkers of Training Responses: A Systems Framework for Exercise Adaptation and Athlete Monitoring

**DOI:** 10.3390/ijms27083601

**Published:** 2026-04-17

**Authors:** Dan Cristian Mănescu, Andreea Voinea, Camelia Daniela Plastoi, Alexandra Reta Iacobini, Alina Anca Vulpe, Ancuța Pîrvan, Corina Claudia Dinciu, Bogdan Iulian Vulpe, Cristian Băltărețu, Adrian Iacobini

**Affiliations:** 1 Department of Physical Education and Sports, Bucharest University of Economic Studies, 010374 Bucharest, Romania; dan.manescu@defs.ase.ro (D.C.M.); corina.dinciu@defs.ase.ro (C.C.D.); adrian.iacobini@csu.ase.ro (A.I.); 2 Sport and Health Department, Faculty of Medical and Behavioral Sciences, Constantin Brâncuși University of Târgu-Jiu, 210135 Targu-Jiu, Romania; camelia.plastoi@e-ucb.ro; 3 Department of Physical Education and Sports, Faculty of Physical Education and Sport, Spiru Haret University, 030045 Bucharest, Romania; alexandra.iacobini@spiruharet.ro; 4 Department of Physical Education and Sports, Technical University of Civil Engineering Bucharest, 020396 Bucharest, Romania; alina.vulpe@utcb.ro (A.A.V.); bogdan.vulpe@utcb.ro (B.I.V.); 5 Department of Physical Education and Sports, Faculty of Humanities, Valahia University of Târgoviște, 130105 Targoviște, Romania; ancuta.pirvan@valahia.ro; 6 Doctoral School, National University of Physical Education and Sport, 060057 Bucharest, Romania; 7 Romanian Bodybuilding and Fitness Federation (FRCF), 030167 Bucharest, Romania

**Keywords:** overtraining syndrome, functional overreaching, non-functional overreaching, multi-omics biomarkers, biomarker panels, multi-omics, athletic monitoring, proteomics, metabolomics, mitochondrial stress, recovery failure index

## Abstract

Exercise adaptation depends on overload that is resolved by recovery, yet the same biology becomes maladaptive when immune, endocrine, metabolic, and muscle-centered stress signals fail to normalize. Exercise-induced maladaptation represents a systems-level failure of biological resolution, with direct relevance to disease-like dysregulation. Functional overreaching, non-functional overreaching, and overtraining syndrome remain difficult to diagnose because no single biomarker provides adequate specificity, temporal stability, or clinical portability. This narrative review synthesizes human and mechanistic evidence across proteomics, transcriptomics, metabolomics, endocrine profiling, extracellular vesicles, and mitochondrial quality-control biology to define the molecular architecture most relevant to athlete monitoring. Across these layers, the most coherent signatures cluster in immune-acute-phase activation, redox-buffering strain, endocrine drift, altered substrate availability, excitation–contraction dysfunction, integrated stress-response signaling, and defects in autophagy–mitophagy and lysosomal remodeling. Three translational elements emerge from this synthesis: a systems-convergence model of recovery failure, a staged biomarker deployment hierarchy, and a provisional recovery failure index. The practical priority is therefore not a solitary marker, but serial phenotype-anchored multimarker panels that connect circulating signals with muscle-centered biology and support decision-making before prolonged recovery failure becomes entrenched.

## 1. Introduction

Exercise adaptation is a controlled biological wager: overload is imposed to disturb homeostasis, yet the intended reward—improved function—appears only if repair, substrate repletion, and neuroendocrine coordination are allowed to catch up. In the applied setting, the same training stimulus may be productive in one athlete and harmful in another because nutrition, sleep, illness burden, psychosocial stress, travel, environmental load, and competition density alter the organism’s capacity to resolve perturbation [1,2]. The central coaching and clinical problem is therefore not whether overload should occur, but when biological strain remains adaptive and when it begins to produce recovery failure.

The continuum linking adaptive overload, functional overreaching (FOR), non-functional overreaching (NFOR), and overtraining syndrome (OTS) is conceptually familiar but operationally difficult. FOR is usually viewed as a deliberately induced, short-lived decrement that resolves after rest and may be followed by supercompensation. NFOR reflects a more persistent decrement with delayed recovery, whereas OTS denotes a prolonged state of underperformance accompanied by fatigue and multisystem disturbance [1,2]. These states are not separated by a single threshold molecule or a single symptom; rather, they emerge from interacting immune, endocrine, metabolic, neural, and muscle-centered processes that unfold across different time scales.

That gap between concept and diagnosis remains the field’s most persistent weakness. Recent reviews and cohort studies continue to emphasize that OTS has no gold-standard diagnostic test and that current practice still relies on performance decline, symptom history, and the exclusion of competing medical explanations [3,4,5,6]. At the same time, the molecular literature has become substantially richer. Proteomics has revealed reproducible immune-related signatures during functional overreaching [7,8]; endocrine studies have highlighted shifts in testosterone, estradiol, catecholamines, lactate, and creatine kinase [9,10]; and mechanistic work has connected excessive loading with altered muscle contractile function, calcium sensitivity, mitochondrial stress signaling, and quality-control failure [11,12,13,14].

This review is built around a translational question that sits squarely at the intersection of molecular exercise biology and sport monitoring: which molecular signatures are most informative for identifying unresolved training stress before the athlete becomes trapped in a long recovery cycle, and how should those signals be staged in practice [4,5,6,15,16,17]? To answer that question, we move from phenotype definition and operational boundaries to immune, endocrine, metabolic, muscle, and mitochondrial domains, and then toward a practitioner-oriented framework in which biomarker interpretation is serial, multimarker, and context-aware rather than reductionist [18,19,20,21]. We also introduce a provisional recovery failure index (RFI) and a deployment hierarchy intended to separate field-ready monitoring from escalation assays and research-stage liquid biopsies.

The review is guided by five explicit questions stated below:

**Q1.** Which molecular programs distinguish productive overload from unresolved maladaptation, and at what point does normal adaptation cease to be biologically efficient?

**Q2.** Which biospecimens and molecular layers are most informative in the early transition from functional overreaching to prolonged recovery failure: routine blood chemistry, endocrine panels, proteomics, metabolomics, or secreted vesicle cargo?

**Q3.** Why have single biomarkers repeatedly failed to generate stable diagnostic thresholds, and why does the biology of training maladaptation favor multimarker interpretation anchored to phenotype and sampling time?

**Q4.** How do mitochondrial stress, redox imbalance, proteostasis, lysosomal signaling, autophagy–mitophagy, excitation–contraction coupling, and inter-organ communication converge to create the muscle-centered phenotype of maladaptation?

**Q5.** How should biomarker studies be designed so that molecular signatures become usable for athlete monitoring rather than remaining mechanistic observations without practical interpretability?

**Figure 1:**
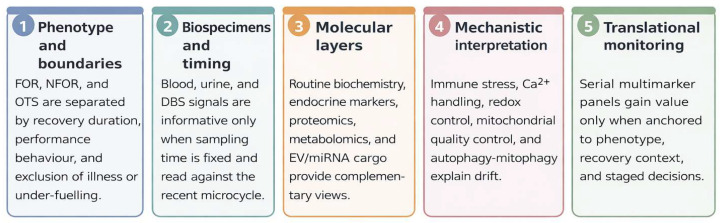
Structure of the review. The manuscript is organized across five linked levels: phenotype and operational boundaries, biospecimens and timing, molecular layers, mechanistic interpretation, and translational decisions. The layered sequence reflects the diagnostic-to-monitoring progression emphasized in current overtraining and athlete-biomarker syntheses [4,6,16].

The five guiding questions define the analytical progression of this review, from distinguishing adaptive versus maladaptive responses to identifying actionable biomarker strategies. This progression is summarized in Figure 1.

Here, we argue that training maladaptation is best understood as a systems-level failure of recovery biology rather than as an extreme of training load alone, a framing that is also compatible with recent conceptual work on oscillatory load, energetic strain, and molecular plasticity [5,17,22]. In this context, excessive exercise stress emerges as a biologically informative model of unresolved molecular and physiological dysregulation. This review introduces three integrative contributions intended to bridge molecular biology and applied athlete monitoring: (1) a systems-convergence model of recovery failure linking multi-domain stress signals to failed biological closure; (2) a staged biomarker deployment hierarchy distinguishing field-ready markers from escalation and research-stage assays; and (3) a provisional recovery failure index (RFI) designed to support serial, phenotype-anchored decision-making. Together, these elements aim to move the field beyond descriptive biomarker lists toward an operational framework that is both mechanistically grounded and practically interpretable.

### Literature Review Strategy and Scope

This narrative review was assembled through structured PubMed searches updated to March 2026 using combinations of the terms ‘functional overreaching’, ‘non-functional overreaching’, ‘overtraining syndrome’, ‘exercise proteomics’, ‘exercise metabolomics’, ‘skeletal muscle transcriptomics’, ‘mitochondrial stress response’, ‘autophagy’, ‘mitophagy’, ‘oxidative stress’, ‘reactive oxygen species’, ‘redox signaling’, ‘Nrf2/Keap1′, ‘extracellular vesicles’, ‘microRNA’, ‘RED-S’, and ‘low energy availability’. Backward citation tracking was used to capture seminal definitions, mechanistic anchors, and contemporary multi-omics studies. The search was designed to prioritize mechanistic relevance and translational interpretability rather than exhaustive study counting.

Priority was given to peer-reviewed human studies in trained or competitive populations, longitudinal or repeated-sampling designs, muscle-biopsy or omics datasets, and reports explicitly linked to performance, fatigue, delayed recovery, or clinical phenotype. Reviews, consensus statements, and foundational physiological papers were retained when they clarified definitions, pathway logic, or methodological constraints. Animal and cell studies were used selectively for mechanistic interpretation where direct human evidence remains limited. The literature was organized into five thematic blocks: (1) conceptual and diagnostic frameworks for FOR, NFOR, and OTS; (2) exercise transcriptomics, proteomics, metabolomics, and sportomics resources; (3) mitochondrial quality control, calcium handling, autophagy, lysosomal signaling, and integrated stress-response biology; (4) extracellular vesicles, exerkines, and circulating microRNAs; and (5) the endocrine–metabolic interface represented by low energy availability and RED-S-related biomarker studies. This was designed as a structured narrative synthesis rather than a formal systematic review, with an emphasis on mechanistic integration and translational relevance. No PRISMA-based screening workflow was applied because the aim was integration and framework building rather than quantitative evidence aggregation.

The evidence base is also asymmetric in depth. Consensus and diagnostic papers provide the operational vocabulary for FOR, NFOR, and OTS, whereas the strongest molecular detail often comes from small overload models, case studies, or mechanistic muscle studies. Large transcriptomic, proteomic, and consortium-scale resources now offer a broader reference frame for what coordinated exercise-responsive biology looks like in humans, but clinically well-phenotyped OTS cohorts remain rare. The synthesis below therefore gives the greatest weight to phenotype-anchored human data and uses preclinical work primarily to interpret pathway directionality rather than to infer clinical thresholds.

Accordingly, the review was structured not only to identify candidate biomarkers of maladaptation, but also to clarify how exercise-induced recovery failure can be interpreted within broader mechanisms of stress integration and biological resolution.

## 2. From Adaptive Overload to Training Maladaptation

### 2.1. Definitions and Operational Boundaries

A useful starting point is to abandon the idea that FOR, NFOR, and OTS are isolated boxes. They are better understood as states distributed along a continuum of biological strain and incomplete recovery. What separates them is not simply symptom severity, but the duration of impaired performance, the speed of recovery after load reduction, and the extent to which signals spread from a local training response to a multisystem pattern involving immune, endocrine, metabolic, autonomic, and psychological domains [1,2,5]. Throughout this review, maladaptation is used as the umbrella term for the transition from productive overload toward unresolved dysfunction; recovery failure refers to the persistence of biological and functional disturbance beyond the athlete’s expected recovery window; and uncoupling denotes loss of the normal alignment between applied load, molecular repair, and performance rebound [1,2,5,16].

The most practical distinction is temporal and functional. In FOR, the athlete tolerates a brief decrement because the subsequent recovery period is built into the plan. In NFOR, recovery takes longer than expected and the athlete begins to lose confidence in the normal relationship between load and rebound. In OTS, the decrement becomes persistent, symptoms broaden, and the athlete no longer behaves like a temporarily fatigued performer but like an organism that has lost normal conditioning responses [9,10]. This loss-of-conditioning concept is important because it moves the discussion away from the simplistic notion that OTS is merely ‘too much training’ and toward the more accurate view that maladaptation emerges from a mismatch between external load and total recovery environment.

**Figure 2:**
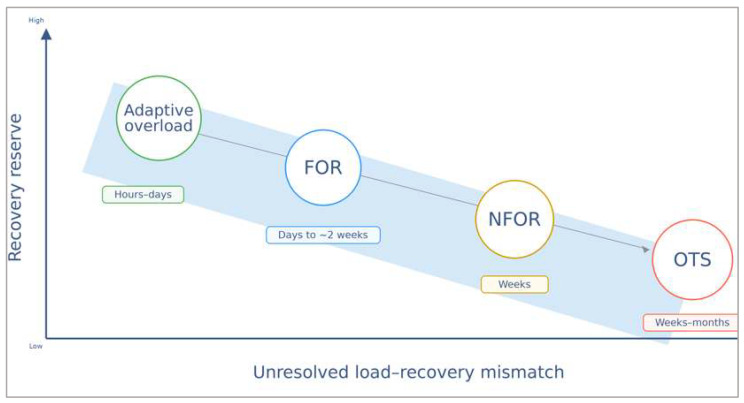
Conceptual schematic continuum from adaptive overload to overtraining syndrome (OTS). States are positioned along a descending band of recovery reserve as unresolved load-recovery mismatch increases from left to right. Circles denote adaptive overload, functional overreaching (FOR), non-functional overreaching (NFOR), and OTS; the labels below indicate pragmatic recovery windows (hours–days, days to ~2 weeks, weeks, and weeks–months). The vertical and horizontal axes are qualitative rather than quantitative and are intended to indicate directional change only. The schematic emphasizes shrinking rebound capacity and progressively greater biological uncoupling across the continuum [1,2,4,5,6].

This progression is summarized schematically in Figure 2, which positions adaptive overload, FOR, NFOR, and OTS along a continuum of decreasing recovery reserve and increasing load-recovery mismatch [1,4,5,6].

Table 1 should therefore be read as an operational continuum rather than as a rigid diagnostic ladder. Its main value is to distinguish a planned, reversible perturbation from a state in which recovery time itself becomes biologically informative.

### 2.2. Limitations of Current Diagnostic Approaches

Three methodological problems keep the field stuck. First, studies rarely capture the full phenotype. A biomarker cannot be interpreted well if the accompanying information on recent load, illness, caloric intake, sleep, menstrual or hormonal status, travel, and psychological stress is poor. Second, many studies examine a single time point even though maladaptation is inherently longitudinal. Third, the biological signal is often anchored to a broad label such as ‘fatigue’ without rigorous performance phenotyping [3,4,5,6]. This heterogeneity is amplified by the fact that studies use different diagnostic criteria, exclusion procedures, performance tests, symptom inventories, and recovery windows to define OTS or related underperformance states, which limit direct comparability across cohorts even when biological trends are directionally similar [3,4,5,6].

These limitations help explain why the literature contains both exciting findings and persistent uncertainty. A marker may be biologically meaningful without being diagnostically decisive. This review therefore treats candidate biomarkers not as standalone tests, but as contributors to a serial interpretation model in which biological data gain meaning only when integrated with the athlete’s recent trajectory.

## 3. Immune and Inflammatory Signatures of Maladaptation

### 3.1. Acute Phase and Innate Immune Signals

Among the most compelling biomarker studies in this space is the proteomic work on functional overreaching by Nieman and colleagues. Using dried blood spot proteomics during a 3-day overload model, the authors identified a 13-protein cluster associated with FOR, most of it linked to the acute phase response and innate immune activation [7]. This study is important not because it solved diagnosis, but because it demonstrated that productive overload leaves a coherent molecular footprint that extends beyond conventional markers such as creatine kinase. More specifically, Nieman et al. [7] quantified 593 dried-blood-spot proteins, observed 60 proteins that rose acutely after day 1 of the overload block (30 immune-related), and then defined a 13-protein FOR cluster on the basis of persistence into recovery day 1 or 2 rather than the immediate post-exercise sample. That recovery-linked cluster included serum amyloid A-4, myeloperoxidase, complement C8 gamma chain, complement C4B, plasma protease C1 inhibitor, inter-alpha-trypsin inhibitor heavy chain H4, alpha-1-acid glycoprotein 2, complement C7, alpha-2-HS-glycoprotein, immunoglobulin kappa and mu constant regions, corticosteroid-binding globulin, and glutaredoxin-1 [7].

The RAAM case study by Merritt and colleagues reinforced that message under an ultra-endurance scenario more consistent with NFOR-like stress. Their targeted proteomic panel highlighted proteins involved in complement activation and the acute phase response, again pointing to immune-related biology rather than a simple muscle-damage narrative [8]. Together, these papers suggest that the blood proteome may detect maladaptive strain earlier and more holistically than traditional single-analyte monitoring. In that case study, blood was sampled 4 weeks, 24 h, and 2 h before the start, twice daily during the race, and again on recovery days 1 and 4. Using a targeted 21-protein panel, the largest increases in the NFOR-like phase were complement C7 (+359%), complement C4-B (+231%), serum amyloid A-4 (+210%), inter-alpha-trypsin inhibitor heavy chain H4 (+191%), and alpha-1-antitrypsin (+188%) [8].

### 3.2. Dysregulated Inflammatory Responses in Maladaptation

Inflammation is not inherently pathological in sport; it is part of normal remodeling. The problem begins when the inflammatory signal is prolonged, poorly resolved, or amplified by non-training stressors. In that situation, the same biological systems that support adaptation may instead contribute to sustained fatigue, altered mood, impaired immune competence, and slower tissue recovery [2,5,15].

This is one reason why isolated cytokine measurements have often disappointed: the signal is timing-sensitive and strongly shaped by the training session that preceded sampling [16,23,24]. Acute phase proteins, neutrophil-derived proteins, complement-related markers, and simple hematological ratios may therefore be more informative when sampled repeatedly and interpreted as a pattern rather than as independent winners or losers [7,8,16]. The practical implication is straightforward: inflammatory biology remains central, but it has to be monitored longitudinally and in context [4,16]. For example, IL-6 behaves mainly as a rapid session biomarker, whereas serum amyloid A and complement-related proteins can remain abnormal across the subsequent 24–48 h, making the latter more useful for recovery-day surveillance [7,16,23,24].

### 3.3. Converging Immune-Proteomic Patterns Across Overload Models

Across the human overload literature, convergence is strongest at the level of immune-proteomic patterning rather than single cytokines. Short overload blocks, ultra-endurance case studies, and clinically phenotyped OTS cohorts all point toward a recurring cluster composed of acute-phase proteins, complement-related biology, neutrophil-associated signals, and altered simple hematology, even though the exact members of the panel differ by platform and recovery window [4,7,8,9,10,16,23]. What appears to generalize is not a universal molecule but a biological direction: when overload ceases to be efficiently resolved, recovery-day blood no longer resembles a transient post-exercise perturbation and instead retains features of innate immune activation. This makes immune-proteomic signatures most useful as trajectory markers that distinguish expected rebound from prolonged recovery failure. These studies also clarify the key timing issue. In Nieman et al., a protein entered the FOR cluster only if it remained elevated on recovery day 1 or 2 and was not merely an immediate post-exercise spike; in Merritt et al., the most informative comparison used prerace baselines against samples from race days 8–9 and recovery day 1 [7,8]. In contrast, lysozyme C, neutrophil elastase, neutrophil defensin 1, S100A12/S100A8, and cathelicidin behaved more like acute load-responsive proteins in the 3-day overload model [7]. Translationally, complement-acute-phase persistence across the next recovery window is therefore more informative for maladaptation than a short-lived cytokine excursion within hours of a hard session.

### 3.4. Sources of Divergence and False-Positive Inflammation

At the same time, the inflammatory literature is heterogeneous for good reasons. Cytokines show narrow kinetic windows, and DBS proteomics and venous plasma do not sample identical biology [7,16,23,24]. The same athlete may also present a different inflammatory profile depending on illness exposure, glycogen status, caloric sufficiency, menstrual or hormonal context, travel stress, and the preceding microcycle [1,4,16,17,25,26]. These factors explain why apparently contradictory studies can still be biologically compatible. For translational purposes, the main lesson is methodological: the field should stop asking whether ‘inflammation’ is present and instead ask whether immune-related signals remain inappropriately elevated for the athlete’s recovery state, phenotype, and sampling window.

Representative sources informing Table 2 include the functional and non-functional overreaching proteomics studies [7,8], the EROS endocrine-biochemical cohorts [9,10], athlete-workload biomarker guidance [16], and energy-availability/substrate-use studies relevant to maladaptation interpretation [25,26].

Read longitudinally, the domains in Table 2 suggest a tiered monitoring logic: inflammatory and endocrine-metabolic layers are first-line candidates, whereas deeper omics signatures become most useful when routine markers and phenotype remain discordant [3,7,8,9,10,16]. These layers are hierarchical rather than additive; most athletes will not require simultaneous interrogation of every domain, and escalation should follow persistent phenotype-biology mismatch rather than analytic curiosity alone, ideally within transparent decision frameworks that remain physiologically interpretable [16,24,27].

## 4. Endocrine, Metabolic, and Muscle-Centered Stress Signatures

### 4.1. Hormonal and Biochemical Patterns

The EROS series remains one of the most informative human programs for understanding the multisystem signature of OTS. In the EROS-BASAL study, OTS-affected male athletes showed lower testosterone and neutrophils, higher estradiol, catecholamines, lactate, and creatine kinase, and a markedly reduced testosterone:estradiol ratio compared with healthy athletes [10]. The key lesson is not that one hormone becomes diagnostic, but that healthy athletic conditioning itself carries a distinct biochemical pattern that can be lost or inverted during maladaptation.

The broader EROS analysis pushed this idea further by framing OTS as a state of ‘paradoxical deconditioning’, in which many athlete-favorable adaptations disappear and the biological profile drifts toward that of nonathletic controls [9]. Complementary endocrine work and related reviews have implicated altered HPA-axis behavior, cortisol awakening responses, abnormal non-exercise stress responses, and contextual disruptors as part of that multisystem picture [28,29,30,31,32]. The EROS-DIAGNOSIS paper then showed why composite tools are attractive: when clinical features were combined with basal hormones and, in some versions, stimulation-test responses, discrimination improved markedly within that cohort [3]. Even if those tools still require broader validation, they underscore a principle that likely applies across all biomarker research in sport: single markers are fragile; structured combinations are more robust.

That endocrine picture also has a fuel-sensing counterpart. Bellinger argued that performance-defined functional overreaching is metabolically heterogeneous and may reflect context-dependent combinations of glycogen stress, low energy availability, and incomplete autonomic recovery rather than a single biochemical state [17]. Recent conceptual work on training–fuel coupling extends that logic by arguing that under-fueling and misaligned nutrient timing can convert an otherwise productive training signal into prolonged recovery failure [33]. In keeping with that idea, Coates and colleagues found that overreached endurance athletes displayed lower continuous-glucose-monitoring-derived glucose and suppressed carbohydrate oxidation during standardized submaximal exercise, suggesting that substrate selection and metabolic flexibility may deteriorate before classical damage markers become dramatic [34,35]. For a molecular review, the implication is important: biomarkers of maladaptation should not be limited to damage molecules, but should also report how the muscle partitions and uses fuel under load.

Low energy availability deserves explicit attention because it can biologically magnify many features that are otherwise attributed to training load alone [17,25,26,36,37,38,39,40,41,42,43,44,45,46,47]. RED-S-related studies consistently show that changes in endocrine tone, glycogen restoration, iron handling, hematological variables, and resting metabolic support can alter the background against which recovery biomarkers are interpreted [25,26,36,41,42,43,44]. In practice, an athlete may therefore present with immune or hormonal signatures suggestive of training maladaptation while the dominant upstream driver is chronic under-fueling [25,26,41,42,43,44]. This is one reason why endocrine panels, ferritin or iron-related markers, CK, and inflammatory readouts should never be interpreted without nutritional context [16,25,26,41,43]. Selective supplementation may support remodeling, but it cannot substitute for the restoration of energy availability and appropriate training–fuel matching [33,48]. This endocrine-metabolic perspective provides the bridge to the next layer of the review, because unresolved substrate stress is one route by which circulating abnormalities become persistent muscle-centered dysfunction.

### 4.2. Muscle and Mitochondrial Pathways as Frontier Biomarker Biology

A second frontier lies deeper in the muscle itself, where contractile, metabolic, redox, and mitochondrial signals begin to converge. Classical muscle-fatigue physiology provides the foundation for this view, because prolonged weakness can emerge from interacting changes in excitation–contraction coupling, metabolite handling, and force production [49,50,51]. Cheng and colleagues emphasized that prolonged low-frequency force depression, altered excitation–contraction coupling, and impaired mitochondrial support for force production may help explain the stubborn sensation of weakness that accompanies chronic recovery failure [11]. More recent human work on intensified training with insufficient recovery linked impaired neuromuscular function to reduced myofibrillar Ca^2+^ sensitivity, providing a mechanistic bridge between ‘the athlete feels flat’ and a measurable cellular defect [12].

Preclinical work is now sharpening that bridge. Watanabe and Wada demonstrated that excessive HIIT-like loading can induce myofibrillar force depression in overreaching, while Sanfrancesco and Hood showed that acute contractile activity is sufficient to activate the mitochondrial integrated stress response and ATF4 signaling [13,14]. These mechanisms are not ready-made field biomarkers, but they are highly relevant because they tell us where the next generation of translational signals may emerge: not only in plasma chemistry, but also in markers that report unresolved contractile and mitochondrial stress.

Concretely, this frontier already has measurable nodes. In intensified training with insufficient recovery, Roussel et al. reported impaired single-fiber myofibrillar Ca^2+^ sensitivity that helped explain reduced neuromuscular function at lower activation frequencies [12]. In the acute contractile-activity model of Sanfrancesco and Hood, CAMKIIalpha and JNK2 were activated about sixfold immediately after stimulation, the phosphorylated-to-total eIF2alpha ratio increased during recovery, and ATF4 and CHOP mRNA each increased by about twofold after 1 h [14]. These data make the mitochondrial layer less abstract: Ca^2+^ sensitivity, p-eIF2alpha, ATF4, CHOP, and related ISR/mitochondrial-stress candidates such as ATF5, GDF15, and FGF21 can be treated as a mechanistic escalation set when first-line blood markers and phenotype remain discordant [12,14].

### 4.3. Redox Biology as the Coupling Layer Between Overload and Recovery

Redox biology sits at the interface between useful adaptation and maladaptive spillover. During exercise, reactive oxygen species are not merely by-products of damage; they act as compartmentalized second messengers that interact with AMPK, p38 MAPK, NF-kB-linked transcription, and programs governing antioxidant defense and mitochondrial remodeling [52,53,54,55,56,57]. A practical way to interpret this layer is through an AMPK–mTOR–SIRT1 regulatory triad: AMPK and SIRT1 coordinate fuel stress and mitochondrial remodeling, whereas mTOR helps determine whether the post-exercise state proceeds toward repair and anabolic closure or remains constrained by unresolved energetic strain [53,56,57,58,59,60,61,62,63,64]. A related systems model proposes that repeated oscillation across AMPK-mTOR signaling and NAD+ economy may help explain how adaptive signaling drifts toward metabolic overdrive when recovery is chronically incomplete [65]. NOX2-derived cytosolic ROS is required for normal GLUT4 translocation and exercise-stimulated glucose uptake, underscoring that redox signaling belongs to adaptation rather than to generic oxidative noise [53]. In human skeletal muscle, exercise to exhaustion rapidly activates the Nrf2/Keap1 axis together with AMPK- and p62-linked regulation, while animal work indicates that p62/SQSTM1 and Nrf2 cooperate to increase antioxidant protein expression in oxidative muscle [54,55].

The translational issue is therefore not whether oxidants rise, but whether redox control re-closes on schedule. Failed recovery is more likely to appear as recovery-day persistence of oxidative pressure, insufficient Nrf2-linked buffering, continued cross-talk with inflammatory and integrated stress-response pathways, and loss of metabolic efficiency, rather than as one abnormal oxidation product [54,55,56,57]. Human data also suggest that higher aerobic fitness is associated with a stronger basal antioxidant milieu in skeletal muscle, so the same absolute oxidative burden can carry different biological meanings across athletes [56]. Redox biology is therefore best interpreted as a context-setting layer that modulates immune, endocrine, and mitochondrial readouts instead of as a stand-alone oxidative-stress assay [57].

**Figure 3:**
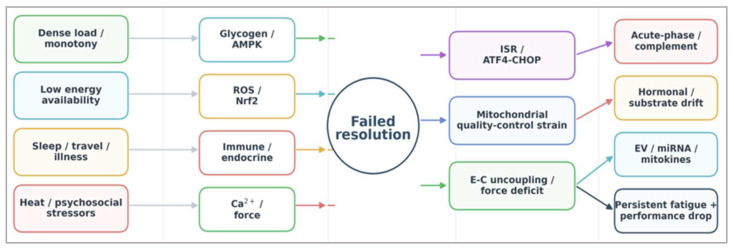
Systems-convergence model of training maladaptation. Contextual stressors feed signal domains involving energy sensing, redox control, immune-endocrine regulation, and contractile/Ca^2+^ handling, which converge on a failed-resolution hub. Persistent activation is then expressed through ISR/ATF4-CHOP signaling, mitochondrial quality-control strain, and excitation–contraction uncoupling, with downstream readouts in blood, secreted factors, and performance phenotype [11,12,13,14,53,54,55,66]. Illustrative signals mapping onto these nodes include glucose/CGM and lactate (energy sensing), acute-phase or complement-related proteins and leukocyte ratios (immune-endocrine spillover), CK or submaximal force behavior (contractile strain), and exploratory GDF15/FGF21 or EV-linked signals (mitochondrial spillover) [7,8,9,10,16,35,67]. Arrows indicate dominant left-to-right routes rather than exclusive one-to-one mappings. Abbreviations: Ca^2+^, calcium; ISR, integrated stress response; ATF4, activating transcription factor 4; CHOP, transcription factor CHOP.

The convergence of these molecular and physiological signals into a unified maladaptive phenotype can be conceptualized as a systems-level failure of recovery integration, as illustrated in Figure 3.

### 4.4. Proteostasis, Lysosomal Remodeling, and Mitophagy

The next molecular layer concerns quality control rather than force output alone. Repeated contractile stress with incomplete substrate repletion can sustain Ca^2+^, AMPK, and redox pressure, promote eIF2alpha phosphorylation, and activate ATF4/CHOP-linked ISR signaling that interfaces with lysosomal remodeling, autophagy–mitophagy flux, and mitochondrial protein-quality control [14,66,67,68,69,70,71,72,73]. In adaptive training, this sequence is brief and restorative; in maladaptation, it remains open long enough that an initially protective program becomes evidence of unresolved organelle stress.

The key distinction is duration and coupling. These pathways are not pathological because they turn on; they become maladaptive when remodeling, clearance, and rebuilding fail to re-synchronize. The resulting phenotype is plausible: excessive proteolysis, inefficient organelle clearance, declining contractile efficiency, and persistent spillover of stress signals into circulation [11,13,66]. This is why markers linked to eIF2alpha-ATF4 signaling, lysosome-autophagy regulation, FOXO-linked protein breakdown, or mitophagy mediators such as BNIP3, BNIP3L/NIX, and PINK1/Parkin remain mechanistically important even before they become field-ready biomarkers.

At finer resolution, mitochondrial distress is shaped by network remodeling, protein import, and inter-organelle contact biology. Exercise responses increasingly implicate mitochondrial dynamics, import-related UPRmt signaling, ER-mitochondria contact sites, and Mfn2-dependent quality-control programs [70,71,72,73,74,75,76,77,78,79,80,81,82,83,84,85,86]. A chronically overloaded fiber does not simply accumulate damage; it can lose the coordination required to segregate dysfunctional mitochondrial domains, maintain proteostasis, and restore excitation–contraction homeostasis after repeated loading bouts [11,12,13,14].

Mitochondrial stress is also not confined to the fiber. Metabolites, vesicles, GDF15, FGF21, and cell-free mitochondrial DNA may carry unresolved organelle stress into circulation and help connect intramuscular dysfunction with low-grade inflammatory and fatigue phenotypes [67]. These signals remain exploratory in sport, but they offer a biologically coherent bridge between tissue-level quality-control failure and blood-based monitoring. The critical distinction is whether these loops close after recovery or remain biologically open, with persistent spillover into circulation. Interpretively, direct human evidence is currently strongest for altered force behavior, Ca^2+^ sensitivity, selected blood/proteomic changes, and substrate-use abnormalities, whereas much of the detailed ISR/ATF4-CHOP, lysosomal-remodeling, and mitophagy circuitry remains derived from mechanistic preclinical studies or limited biopsy-supported datasets [11,12,13,14,35,66,67,68,69,70,71,72,73].

**Figure 4:**
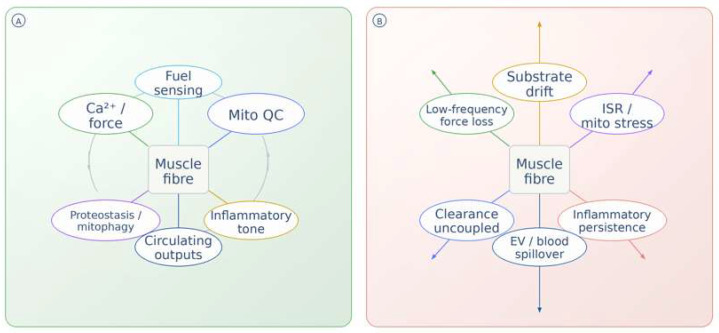
Closed-loop versus open-loop remodeling in skeletal muscle. (**A**) Resolved adaptation, where fuel sensing, force handling, mitochondrial quality control, proteostasis-mitophagy, inflammatory tone, and circulating outputs re-equilibrate around the muscle fiber after recovery. (**B**) Persistent maladaptation, where the same network remains engaged but opens outward into substrate drift, ISR/mitochondrial stress, inflammatory persistence, impaired clearance, force loss, and extracellular/blood spillover [11,12,13,14,66,67,68,69,73]. Representative outputs of loop closure include normalization of symptoms, performance, and first-line blood markers, whereas open-loop persistence may retain CK/lactate drift, acute-phase or complement signals, altered glucose handling, and exploratory mitochondrial-stress or EV readouts [7,8,10,16,35,67]. Identical geometry across panels emphasizes altered coupling and failure of loop closure rather than a wholly different pathway set. Abbreviations: ISR, integrated stress response. Arrows indicate directional relationships, while colors distinguish functional states (resolved vs. persistent maladaptation) and related process groupings.

The distinction between resolved adaptation and persistent maladaptation can be understood as a shift from closed-loop to open-loop biological regulation, as illustrated in Figure 4.

### 4.5. Extracellular Vesicles, microRNAs, and the Exercise Secretome

EVs and circulating microRNAs are attractive because they may report tissue stress before routine resting markers diverge [87,88,89,90], but they are also among the most pre-analytically fragile layers in exercise biology [91,92,93,94]. Exercise modality, sampling window, isolation strategy, storage, matrix choice, and hemolysis can all reshape the signal [90,93]. For maladaptation research, this means that EV or miRNA data are best treated as biologically rich second-line layers rather than as frontline diagnostic tests [24,90,93,95].

Their biological appeal is strong. Acute heavy resistance exercise altered 34 EV microRNAs with predicted targets in the IGF-I, STAT3, PPAR, JAK/STAT, ERK/MAPK, AMPK, mTOR, and PI3K/AKT pathways, placing EV cargo at the intersection of anabolic signaling, metabolism, and immune regulation [96]. Exercise to exhaustion likewise increased urinary EV abundance and altered EV-microRNA cargo with links to PI3K-Akt, MAPK, and insulin signaling [95].

These findings support a simple translational rule: EV and microRNA layers are most useful when first-line monitoring remains abnormal or biologically discordant after adequate deload, and only when pre-analytical control is strict. Used in that position, they may function as liquid biopsies of unresolved tissue stress rather than as universal screening tools.

Key mechanistic sources informing Table 3 include work on prolonged weakness and excitation–contraction dysfunction [11], impaired myofibrillar Ca^2+^ sensitivity with intensified training [12], excessive HIIT-like overreaching and muscle-force depression [13], acute mitochondrial ISR activation [14], lysosomal-autophagy remodeling [66,73], and exploratory EV/secretome studies [67,95,96].

The importance of Table 3 is mechanistic rather than diagnostic. Its added timing and feasibility cues are intended to show when a signal is most likely to emerge and how realistically it can be captured in the field. As the signal moves from fuel sensing toward organelle quality-control failure, accessible blood readouts still need to be interpreted alongside performance phenotyping, and in research settings, tissue-level assays [11,12,13,14,21,24,66,97].

Representative sources supporting the stage-linked logic in Table 4 are workload-biomarker guidance [16], overload proteomics studies [7,8], endocrine/biochemical OTS cohorts [9,10], fuel-stress work [35], and mechanistic redox-mitochondrial studies [53,54,55,66,67,73].

Operationally, Table 4 shifts the question from whether a marker rises after hard training to whether that rise resolves on schedule for the individual athlete and context. The timing and feasibility cues are intended to guide when monitoring should simply be repeated, when deload should precede escalation, and when research-stage biology is unlikely to be useful in routine practice.

## 5. Multi-Omics Integration for Next-Generation Biomarker Panels

### 5.1. Superiority of Multimarker Panels over Single Biomarkers

Emerging large-scale resources are beginning to show what a mature molecular reference frame for exercise actually looks like. Meta-analytic transcriptomic atlases, consortium-scale molecular mapping initiatives, and contemporary multi-omics resources now define conserved early-response modules, modality-dependent remodeling programs, and tissue-scale signatures across human skeletal muscle [18,19,20,21,98,99,100,101,102,103]. For maladaptation research, the implication is decisive: a biomarker panel should not be judged against a generic exercise response, but against the molecular direction expected for the specific loading paradigm, recovery window, tissue source, and athlete phenotype under study [20,21,97,100,101,104]. In practical terms, the near-term role of omics is not discovery alone, but calibration of the biological direction expected for a given load-recovery context [18,19,20,21,100,101].

The attraction of proteomics is not merely that it can measure more molecules. Its real value is that it captures coordinated biology. The FOR and NFOR studies already discussed suggest that maladaptation is expressed as a network-level shift involving acute phase proteins, innate immunity, complement activity, and broader immunometabolic processes [7,8]. This logic aligns well with more recent thinking in the OTS literature, where composite systems and complex-systems models have replaced the search for a single universal biomarker [3,4,5].

A practical multimarker example is to combine a recovery-day proteomic signal (e.g., serum amyloid A-4, myeloperoxidase, and complement C4B/C7/C8-related persistence), an endocrine-biochemical layer (testosterone:estradiol ratio, catecholamines, CK, lactate), and a metabolomic/fuel-use layer (CGM-derived glucose drift, carbohydrate-oxidation behavior, and broader metabolite signatures spanning lactate, amino acid, lipid, or TCA-cycle intermediates) against the athlete’s performance and symptom baseline [7,8,9,10,34,35]. In that hierarchy, proteomics contributes the coordinated immune-acute-phase direction, metabolomics captures substrate stress and metabolic flexibility, and transcriptomic or EV-linked layers are best viewed as escalation tools for unresolved cases rather than as first-line screening. This is the level of multi-omics integration meant by multimarker panels in the present review.

For a translational review intended for practitioners, this point is essential. Coaches do not need more isolated numbers; they need decision support, and any analytic layer introduced into monitoring should remain interpretable enough for clinicians and performance staff to understand why a profile is escalating rather than behaving as an opaque classifier [6,7,8,16,27]. A useful biomarker panel should therefore satisfy four criteria: it should detect deviation from the athlete’s own baseline, correlate with performance or recovery, remain interpretable across repeated measurements, and add value beyond symptom monitoring alone. Multi-omics strategies are attractive only to the extent that they satisfy those applied criteria [18,19,20,21,24,97,100].

Time resolution is a major reason why omics may succeed where isolated biomarkers fail. A meta-analysis of 43 exercise transcriptomic studies showed that acute and long-term responses are transcriptionally distinct, with many genes displaying narrow time windows and strong modulation by modality, age, and other cohort characteristics [101]. For maladaptation research, this means that an apparently inconsistent biomarker may simply have been sampled at the wrong biological moment. Omics should therefore be planned around kinetic questions: what rises immediately after overload, what remains elevated after 24–72 h, and what becomes chronically reset across a training block.

Equally important, overtraining biology should not be modeled as the mirror image of training adaptation. Comparative transcriptomic analyses show that decreased and increased skeletal-muscle loading activate qualitatively different transcription-factor networks rather than simple inversions of the same program [105]. Epigenetic work further indicates that greater physiological and metabolic load can reshape DNA methylation together with genes linked to AMPK/MAPK signaling, VEGFA, NR4A1, NR4A3, and PPARGC1A [106]. Conceptual work on training-load oscillation and epigenetic plasticity likewise supports the view that maladaptation reflects altered remodeling dynamics rather than a simple linear excess of load [22]. This supports a systems view in which maladaptation reflects failed remodeling, not merely an exaggerated version of a normal acute response. The near-term challenge is therefore not to generate ever larger candidate lists, but to identify minimal multimarker combinations that remain stable, affordable, and decision-relevant in real athlete monitoring. Omics will not replace monitoring, but it will redefine its resolution.

### 5.2. Standardization, Phenotyping, and Study-Design Priorities

The fastest way to create noisy biomarker literature is to ignore pre-analytical control. Sampling time, fed or fasted status, matrix choice, recent exercise, menstrual or hormonal context, altitude or heat exposure, travel, assay platform, and recent infection can all distort interpretation [1,4,5,16,33]. This is one reason the field still lacks portable decision thresholds despite decades of interest. Recent athlete-biomarker guidance has also stressed the importance of analytical variation, meaningful minimal change, and the athlete’s own reference range when serial values are used for workload management [16]. For emerging analytes such as extracellular vesicles or microRNA panels, these concerns are magnified rather than reduced [90,93,95].

A mature biomarker framework for training maladaptation must therefore be standardized at three levels: the athlete context, the sampling procedure, and the phenotype anchor [16,24,90,93,95]. Serial morning samples during defined training phases, repeated use of the same assay platform, matrix-specific handling protocols, and pairing of biomarker data with symptom and performance information should be considered minimal quality standards for future studies [7,8,16,24,90,93,95]. Without that discipline, even biologically important molecules will remain translationally weak [16,90,93].

Key pre-analytical and analytical priorities for biomarker studies in training maladaptation are summarized in Table 5.

These pre-analytical constraints explain much of the field’s instability. Without fixed sampling windows, consistent matrix handling, and phenotype anchors, even biologically valid signals will continue to appear contradictory across cohorts [1,4,5,16,90,93]. External validity is further limited by small overload models, mixed training backgrounds, male-dominant samples, and the scarcity of longitudinal cohorts with clinically well-phenotyped OTS. Until larger datasets are available, the field should prioritize within-athlete tracking, staged escalation rules, and explicit capture of illness exposure, energy availability, hormonal context, and recovery environment over universal cut-offs.

## 6. Discussion: From Molecular Signals to Phenotype-Anchored Monitoring

The present synthesis extends beyond descriptive molecular mapping by proposing an integrated, phenotype-anchored framework that links mechanistic biology to staged monitoring and decision-making in athletes.

### 6.1. Answer to Q1: When Productive Overload Becomes Unresolved Maladaptation

The transition from productive overload to maladaptation is best identified by failed recovery, not by the peak acute response. Productive overload produces temporally appropriate perturbation, whereas unresolved maladaptation is characterized by recovery-day persistence of immune-redox activation, loss of athlete-like endocrine-metabolic patterning, and delayed return of function [1,2,3,4,5,6,9,10,16]. This distinction is biologically important because OTS is not simply the far-right tail of training load; it is a state in which the normal coupling between overload, repair, substrate restoration, and performance rebound begins to break down [5,11,15,16,17,65]. The field should therefore stop asking whether inflammation, hormonal drift, or mitochondrial stress occur after hard training; they do. It should instead ask whether these signals remain inappropriately open for the athlete’s expected recovery window [4,5,6,16,23,24]. In practice, the timing of signal persistence is often more informative than the absolute height of the preceding excursion [16,23,24].

### 6.2. Answer to Q2: Which Biospecimens and Molecular Layers Are Most Informative

For near-term monitoring, the most informative first-line biospecimens remain serial blood measurements interpreted against phenotype: simple hematology, acute-phase or innate-immune proteins, and selected endocrine variables [3,6,7,8,10,16], together with metabolic readouts linked to substrate use or contractile strain [35,107,108,109,110,111,112]. These layers are imperfect, but they are accessible, repeatable, and already connected to recovery duration or underperformance. Operationally, biomarker layers are most useful when tiered by deployment readiness: field-ready serial anchors, escalation assays used when first-line biology and phenotype remain discordant, and research-stage liquid biopsies or tissue-centered readouts. Proteomics becomes especially valuable when routine markers look equivocal because it captures coordinated immunometabolic directionality rather than isolated analytes [7,8,21,24,104]. In contrast, EV cargo, circulating microRNAs, and tissue-centered stress markers should currently be viewed as second-line or research-stage layers that help explain persistent or discordant cases rather than replace routine monitoring [21,66,90,93,95,97]. Where under-fueling or impaired remodeling is suspected, nutritional correction and training-fuel realignment should precede deeper testing; targeted supplementation may support tissue adaptation, but it does not substitute for restoration of energy availability [33,48].

One pragmatic field configuration is a tiered panel that pairs symptom/performance anchors with CBC-derived leukocyte shifts, an acute-phase or complement-oriented readout, CK/lactate, selected endocrine measures such as the testosterone:estradiol ratio, and fuel-use readouts such as interstitial glucose or carbohydrate-oxidation behavior; targeted proteomic and metabolomic panels then become escalation layers when recovery remains unresolved [7,8,9,10,16,34,35]. Methodologically, first-line analytes are captured by automated hematology, clinical chemistry, and immunoassays, whereas escalation proteomics and metabolomics rely primarily on LC-MS/MS or NMR platforms and EV/miRNA layers on standardized vesicle isolation plus RT-qPCR or sequencing workflows [16,21,24,90,93,95,98,99,100,101,102,103,104,105,106,107,108,109,110,111,112].

### 6.3. Answer to Q3: Why Single Biomarkers Fail and Why Multimarker Interpretation Is Necessary

Single biomarkers fail repeatedly for three converging reasons: the phenotype is heterogeneous, the kinetics are time-sensitive, and the same analyte can be shifted by illness, low energy availability, sleep disruption, travel, sex-hormonal context, or the preceding microcycle [1,4,5,6,16,25,26,101]. A biologically meaningful marker can therefore be diagnostically weak when removed from timing and context. This is why the most robust framework is a phenotype-anchored multimarker model in which no analyte is interpreted alone and no value is read without the baseline, recent load, symptoms, and recovery duration [3,4,5,6,16,24]. In practice, multimarker panels do not eliminate uncertainty; they refine it by showing whether the athlete is re-closing the recovery loop or drifting into persistent uncoupling [5,16,24].

A critical implication follows: much of the existing literature is not contradictory but under-standardized. Small cohorts, single time points, broad labels such as fatigue, and poor control of recovery biology have produced a literature rich in mechanism yet weak in thresholds [4,16,23,90,93,113,114,115]. Future work should therefore privilege within-athlete serial designs over cross-sectional threshold hunting.

### 6.4. Answer to Q4: How Muscle-Centered Stress Programs Converge

The muscle-centered phenotype of maladaptation appears to emerge when excitation–contraction strain, redox signaling, mitochondrial ISR activation, and autophagy–mitophagy control remain engaged longer than recovery can absorb [11,12,13,14,56,57,66,68,71]. In that state, the athlete may present clinically with disproportionate weakness, altered submaximal force behavior, abnormal substrate selection, and persistent spillover of stress signals into circulation even before classical resting damage markers become dramatic [11,12,13,14,35]. This helps explain why blood-based monitoring remains necessary but incomplete: the decisive biology may originate in muscle, while the measurable field signal is only a downstream echo of incomplete organelle and contractile recovery [21,24,67,81,97].

Within this hierarchy, EV and microRNA layers have a plausible translational niche. They are best deployed when first-line monitoring remains abnormal or biologically discordant after adequate deload, and only under tightly standardized sampling, hemolysis control, matrix handling, and platform consistency [90,93,95]. Used in that way, they may function as second-line liquid biopsies of unresolved tissue stress rather than as front-line screening tools.

### 6.5. Answer to Q5: Study-Design Priorities and a Provisional Recovery Failure Index

The next generation of biomarker studies should be built around dense longitudinal phenotyping, fixed sampling windows, and preplanned escalation from accessible markers to deeper omics layers. Energy availability, chrono-nutritional timing, and hormonal context should be captured explicitly rather than treated as background noise [21,22,23,24,25,26,90,91,92,93,94,95,96].

As a practical synthesis, we propose a provisional recovery failure index (RFI) as an implementation logic rather than a diagnostic threshold. Let phenotype deviation = P, immune-hematological persistence = I, endocrine-metabolic drift = E, and unresolved discordance requiring escalation = D; each domain is scored 0–2 points, giving RFI_raw = P + I + E + D (range 0–8) and, when preferred for dashboards, RFI% = (RFI_raw/8) × 100. The RFI is intended as a conceptual implementation tool to structure serial decision-making rather than as a validated diagnostic instrument. The score is intentionally unweighted to preserve interpretability and to avoid implying false precision before prospective calibration. Future work should test weighted, probabilistic, or Bayesian variants only if they improve discrimination without sacrificing physiological transparency.

The score is intentionally serial and athlete-specific rather than cross-sectional. P can be graded from repeated performance loss, unusual fatigue, or delayed restoration of training tolerance; I from serial acute-phase or leukocyte-based abnormalities; E from loss of the athlete’s expected hormonal or substrate-use profile; and D from persistent mismatch between symptoms, performance, and first-line biology despite deload and context correction. Importantly, the RFI should not be interpreted as a threshold-based classifier, but as a trajectory-sensitive index reflecting whether recovery processes are re-closing over time. A rising score suggests loss of recovery-loop closure, whereas a falling score during deload supports a return toward adaptive overload. Conceptually, the domains are integrated directionally rather than as exchangeable abnormalities: points are added when a variable remains persistently elevated, suppressed, or discordant in the maladaptive direction for that athlete and sampling window, not merely when it differs from population reference ranges [16,24].

For clinicians and performance staff, the operational message is straightforward: first, re-check the context before interpreting biology; second, repeat first-line markers before escalating to deeper assays; and third, treat persistent discordance as a trigger for broader clinical evaluation rather than as proof of OTS by itself [3,5,16,24].

**Figure 5:**
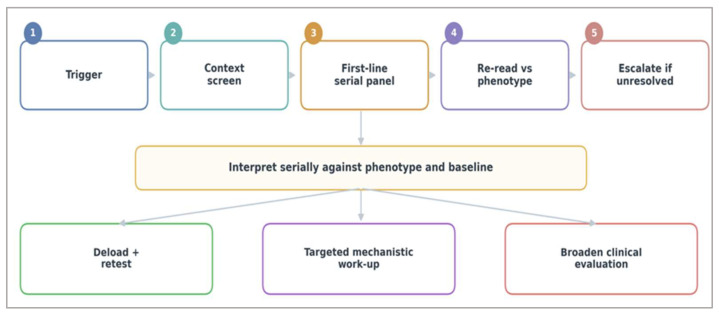
Phenotype-anchored workflow for biomarker-guided monitoring. The sequence proceeds from trigger to context screen, first-line serial panel, re-reading against phenotype, and escalation only when recovery remains unresolved. Management branches are deload and re-test when biology and function improve together, targeted mechanistic work-up when serial biology remains abnormal despite adequate recovery, and broader clinical evaluation when the pattern is discordant, severe, or suggestive of competing diagnoses. The workflow prioritizes serial interpretation against the baseline rather than isolated abnormal values [16,24,33]. Numbers (1–5) indicate the sequential steps of the workflow, arrows represent directional flow between steps, and colors are used to visually distinguish stages and decision pathways without implying quantitative differences.

This systems-level interpretation can be translated into a structured monitoring logic that links biological signals to practical decision-making, as outlined in Figure 5.

### 6.6. A Pragmatic Deployment Hierarchy for Real-World Monitoring

For applied monitoring, candidate biomarkers are most useful when separated by deployment readiness rather than placed in one undifferentiated list. Table 6 groups them into field-ready serial anchors, escalation assays used when first-line biology and phenotype remain discordant, and research-stage liquid biopsies or tissue-centered readouts. The purpose of this hierarchy is to reduce both false reassurance and overtesting: most athletes should be followed with repeated low-burden measures, whereas deeper omics layers should be reserved for persistent, atypical, or mechanistically unclear cases [16,21,24,27,90,93,97]. Interpretable analytic frameworks may help operationalize this hierarchy without turning multimarker monitoring into an opaque black box.

**Figure 6:**
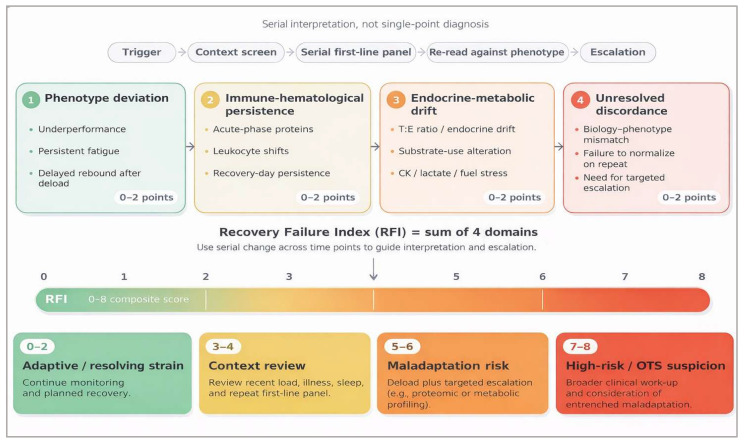
Provisional recovery failure index (RFI) for staged decision support. Four domains—phenotype deviation, immune-hematological persistence, endocrine-metabolic drift, and unresolved discordance—re each scored 0–2 points, yielding an intentionally unweighted serial 0–8 composite index. The lower bands indicate pragmatic action zones: 0–2, adaptive or resolving strain; 3–4, context review and repeat first-line panel; 5–6, deload plus targeted escalation; and 7–8, broader clinical work-up and consideration of entrenched maladaptation. The figure is intended as a serial decision aid rather than a universal diagnostic threshold [3,5,16,24,27]. Colors represent graded risk levels, from adaptive/resolving states (green) to higher-risk maladaptive states (orange–red).

Figure 6 integrates this hierarchy with the provisional RFI. In practice, 0–2 points suggest adaptive or resolving strain, 3–4 points justify context review and repeat first-line sampling, 5–6 points support deload plus targeted escalation, and 7–8 points justify broader clinical work-up for OTS mimics or entrenched maladaptation. The value of the RFI is not that it supplies a universal diagnostic threshold, but that it forces serial interpretation across phenotype, immune-hematological persistence, endocrine-metabolic drift, and unresolved biological discordance.

To operationalize this framework, we propose a provisional recovery failure index that integrates phenotype and molecular signals into a unified decision-support construct, as illustrated in Figure 6.

### 6.7. Limitations

This review has several limitations. First, the evidence base remains heterogeneous in phenotype definition, sampling density, sport discipline, and translational depth, which limits direct comparison across studies. Second, clinically well-phenotyped longitudinal cohorts of true OTS remain rare, so several mechanistic inferences still rely on overload models, case studies, or pathway-focused preclinical work rather than large prospective athlete datasets. Third, many emerging omics, EV, and microRNA signals remain sensitive to pre-analytical variability, assay platform, and sampling timing, which constrains immediate field deployment. Fourth, the provisional RFI proposed here is a conceptual implementation framework and requires prospective validation, calibration against athlete-specific baselines, and testing across sex, sport type, and energy-availability contexts. Fifth, reverse causality remains a major unresolved problem: some molecular profiles may reflect pre-existing vulnerability, chronic low energy availability, illness burden, or constitutional differences in recovery capacity rather than maladaptation induced by training itself. Accordingly, the present synthesis should be read as a phenotype-anchored translational framework rather than as a source of universal diagnostic cut-offs.

## 7. Conclusions

Three conclusions emerge from the evidence reviewed here. Importantly, this review not only summarizes existing evidence but also reorganizes it into a systems-level, operational framework for the early detection of recovery failure.

First, overtraining is not a state of excess load, but a state of failed biological closure. Functional overreaching, non-functional overreaching, and overtraining syndrome are therefore best understood as progressively unresolved recovery biology, defined by convergence among immune, endocrine, metabolic, redox, and muscle-centered stress programs.

Second, the most useful biomarker architectures will be serial, phenotype-anchored, multimarker, and tiered by deployment readiness. A staged panel should begin with performance and symptom anchors plus accessible immune, endocrine, and metabolic readouts, escalate to targeted proteomic or metabolomic assays when recovery remains unresolved, and reserve EV or tissue-centered layers for refractory or research-stage questions. Such panels should privilege within-athlete baselines over cross-sectional thresholds, and the timing of signal persistence should usually carry more interpretive weight than the absolute magnitude of a single abnormal value. Within that logic, a provisional RFI offers a pragmatic way to summarize whether the recovery loop is closing across serial time points. At present, the RFI should be viewed as a translational framework to guide structured monitoring rather than as a standalone diagnostic tool.

Third, future studies should explicitly connect tissue biology to field monitoring. Longitudinal cohorts with dense sampling, explicit control of energy availability and sex-hormonal context, and muscle-to-blood integration are needed to validate practical tools such as the provisional RFI, test tiered escalation rules, and determine whether ISR, calcium-handling, mitophagy, and secretome signals improve decision-making in real athletes. The practical goal is earlier recognition of an open recovery loop before prolonged maladaptation becomes entrenched.

## Figures and Tables

**Table 1:** Operational continuum linking productive overload to overt training maladaptation.

Practical Interpretation	Dominant Biological Picture	Recovery Window	Performance Profile	State
Expected training response	Transient stress signaling matched by recovery	Hours to a few days	Stable or improving	Adaptive overload
Can be planned	Reversible immune-proteomic perturbation with retained adaptive capacity	Days to about 2 weeks	Short-term decrement	Functional overreaching (FOR)
Requires deload and follow-up	Multidomain disturbance and delayed normalization	Weeks	Persistent decrement	Non-functional overreaching (NFOR)
Clinical work-up and staged rebuild	Endocrine, immune, metabolic, and clinical loss of conditioning	Many weeks to months	Long-lasting underperformance	Overtraining syndrome (OTS)

Note: FOR, functional overreaching; NFOR, non-functional overreaching; OTS, overtraining syndrome. The states shown are pragmatic categories along a continuum rather than discrete biological entities, and recovery windows are approximate and influenced by sport discipline, competition density, energy availability, sleep, illness burden, and other recovery conditions [1,2,4,5,6].

**Table 2:** Candidate biomarker domains most relevant to the detection of training maladaptation.

Evidence Base	Interpretation Caveat	Typical Pattern in Maladaptation	Candidate Signals	Domain
Moderate human	Strongly timing-dependent; recent illness can mimic the pattern	Signal persistence or exaggerated recovery-day elevation	Acute phase proteins, complement-related proteins, neutrophil-derived proteins, leukocyte ratios	Immune/ inflammatory
Moderate human	Basal values may appear ‘normal’ relative to reference ranges	Loss of athlete-like conditioning profile	Testosterone, estradiol, testosterone:estradiol ratio, catecholamines	Endocrine
Moderate human	Session design and training type influence direction and magnitude	Disproportionate metabolic or contractile strain	Lactate, creatine kinase, submaximal force measures, Ca^2+^ sensitivity-related surrogates	Metabolic/ muscle
Emerging human	Platform harmonization is still limited	Composite shifts outperform single molecules	Targeted protein panels, metabolite panels, integrated signatures	Proteomics/ multi-omics
Strong applied	Biomarkers are weak if not tied to function	Necessary for phenotype anchoring	Performance tests, symptom scores, perceived effort, recovery duration	Clinical anchor

Note: The domains are presented as serial monitoring layers rather than standalone diagnostic tests. Evidence-base labels reflect translational maturity, not universal diagnostic thresholds. Ca^2+^, calcium. All signals should be interpreted against recent load, illness exposure, energy availability, and the athlete’s symptom and performance trajectory [7,8,9,10,16,24].

**Table 3:** Muscle-centered molecular pathways relevant to training maladaptation, their translational readouts, earliest detection window, and field feasibility.

Domain	Key Readouts	Meaning in Maladaptation	Biospecimen /Maturity	First Signal	Field Feasibility
Fuel sensing/ energetic stress	Glucose, lactate, AMPK-linked signatures	Reduced metabolic Flexibility	Blood or CGM; moderate	Session-24 h	High
Ca^2+^ handling /contractile apparatus	Ca^2+^ sensitivity, force-frequency shift	Cellular basis of the ‘flat’ phenotype	Advanced physiology or biopsy; low	24–72 h	Low-med.
Redox-inflammatory persistence	Acute phase proteins, complement, MPO, ROS/RNS-related tone	Failure to resolve innate immune activation	Plasma, serum, DBS; moderate	Recovery day-72 h	Medium
Mitochondrial ISR/quality control	eIF2alpha-ATF4, CHOP, ATF5, GDF15, FGF21	Mitochondrial stress with loss of resilient remodeling	Biopsy plus exploratory blood markers; low	24 h-block accumulation	Low
Autophagy-lysosome /mitophagy	TFEB/TFE3, BNIP3, BNIP3L/NIX, PINK1, Parkin	Organelle turnover uncoupled from effective recovery	Muscle tissue, EV cargo, targeted assays; low	Block accumulation	Low
Secretome /extracellular vesicles	EV abundance, CD9/CD63/CD81, EV-microRNA panels	Intramuscular stress converted into liquid-biopsy signals	Plasma or urine EVs; low	Hours-24 h	Low

Note: Evidence-base shorthand: Mod., moderate human evidence; Early + mech., early translational evidence supported by mechanistic studies; Explor., exploratory. CGM, continuous glucose monitoring; DBS, dried blood spot; MPO, myeloperoxidase; ROS/RNS, reactive oxygen/nitrogen species; eIF2alpha, eukaryotic initiation factor 2 alpha; ISR, integrated stress response; ATF4/ATF5, activating transcription factors 4 and 5; CHOP, C/EBP homologous protein; GDF15, growth differentiation factor 15; FGF21, fibroblast growth factor 21; TFEB/TFE3, transcription factors EB and E3; BNIP3L/NIX, BCL2 interacting protein 3-like/NIP3-like protein X; PINK1, PTEN-induced kinase 1; EV, extracellular vesicle. Field feasibility is a pragmatic estimate of how realistically a signal can be repeated in routine athlete monitoring. Most readouts below the redox-inflammatory layer remain research-stage and require longitudinal validation in well-phenotyped athlete cohorts [11,12,13,14,24,66,67,73,95,96].

**Table 4:** Stage-linked monitoring logic linking load-recovery mismatch to biomarker-detectable maladaptation, with timing and field-feasibility cues.

Stage	Core Molecular Logic	Preferred Readouts	Timing/ Feasibility	Monitoring Use
Productive overload	Transient AMPK, catecholamine, and substrate stress that resolves on schedule	Lactate or CGM trends; session CK/protein shifts	Session-24 h /high	Expected rebound
Delayed immune-redox resolution	Acute-phase/complement persistence with emerging Nrf2/Keap1 buffering strain	Serial proteomics, leukocyte ratios, exploratory oxidative panels	24–72 h /medium	Context review
Contractile-mitochondrial inefficiency	Reduced Ca^2+^ sensitivity with ISR activation and rising mitochondrial stress tone	Submaximal force tests, targeted blood markers, biopsy in research	48 h-block /medium	Explains prolonged weakness
Quality-control failure	TFEB/TFE3, BNIP3 and BNIP3L/NIX, PINK1/Parkin, and lysosomal-autophagic uncoupling	Tissue markers, EV cargo, targeted assays	Block-prolonged recovery /low	Mechanistic escalation
Systems phenotype of maladaptation	Endocrine drift plus symptom-performance uncoupling across tissues	multimarker panel plus phenotype anchors	Weeks to months /medium	Deload + clinical work-up

Note: The cascade is conceptual and stage-based, with overlap between adjacent states; it translates molecular direction into serial monitoring logic rather than a fixed temporal sequence or rigid diagnostic threshold. AMPK, AMP-activated protein kinase; CGM, continuous glucose monitoring; CK, creatine kinase; Nrf2/Keap1, nuclear factor erythroid 2-related factor 2/Kelch-like ECH-associated protein 1; ISR, integrated stress response; TFEB/TFE3, transcription factors EB and E3; BNIP3L/NIX; PINK1, PTEN-induced kinase 1; EV, extracellular vesicles. Field feasibility refers to routine repeated use in applied monitoring rather than mechanistic importance [16,24,53,54,55,66,67,73].

**Table 5:** Pre-analytical and analytical priorities for future biomarker studies in training maladaptation.

Risk if Ignored	Recommended Standardization	Why it Matters	Variable
False between-session differences	Use fixed morning or fixed post-exercise windows	Many signals are sharply time-sensitive after exercise	Sampling window
Mislabeling normal overload as pathology	Log training load, monotony, and competition density	Biomarkers reflect the preceding microcycle, not just chronic status	Recent load history
Confounded interpretation	Record core recovery stressors at each sampling point	Sleep, travel, illness, and energy availability modulate the same biology	Recovery environment
Reduced reproducibility	Document cycle phase, contraceptive use, or hormonal treatment where relevant	Endocrine signals vary with biological and pharmacological context	Hormonal context
Poor comparability	Use the same matrix and platform within a study or program	Panel composition and absolute values vary by method	Assay platform
Clinically weak conclusions	Pair sampling with symptoms and performance metrics	Biomarkers are meaningful only when tied to function	Phenotype anchor

Note: This checklist summarizes the minimum pre-analytical and analytical controls for serial biomarker studies in athletes. Standardization is most informative when repeated within the same athlete, using the same sampling window, biospecimen matrix, and assay platform, and when interpreted alongside recent load, symptoms, performance, and recovery status [16,24,33,90,93,95].

**Table 6:** Provisional deployment hierarchy for biomarker-guided monitoring of training maladaptation.

Tier	Primary Purpose	Representative Readouts	Practical Trigger to Advance
Field-ready serial anchors	Detect loss of expected rebound in routine monitoring	Performance tests; symptom/recovery scores; simple hematology; acute-phase or innate-immune proteins; CK/lactate; selected endocrine-metabolic measures; CGM trends	Advance when underperformance persists or serial biology fails to normalize after deload/context correction
Escalation assays	Clarify discordance between phenotype and first-line markers	Targeted proteomics; richer metabolite panels; submaximal force testing; targeted redox or contractile surrogates	Advance when first-line data remain abnormal on repeat or a mechanistic explanation is needed
Research-stage/ tissue-centered	Investigate unresolved cases; link circulating signals to muscle biology	EV cargo; microRNA panels; mitochondrial-stress candidates; biopsy-linked ISR/mitophagy readouts	Use in prospective studies or specialist work-ups, not for frontline screening

Note: The hierarchy is designed for serial interpretation, not one-off threshold hunting. Movement across tiers should be driven by persistent phenotype-biology discordance, delayed recovery, or competing diagnostic uncertainty rather than by isolated abnormal values [16,21,24,27,90,93]. CK, creatine kinase; CGM, continuous glucose monitoring; EV, extracellular vesicle; ISR, integrated stress response.

## Data Availability

No new data were generated or analyzed in this study. Data sharing is not applicable to this article.

## Abbreviations

AMPK	AMP-activated protein kinase
ATF4	Activating transcription factor 4
CGM	Continuous glucose monitoring
CHOP	C/EBP homologous protein
CK	Creatine kinase
EVs	Extracellular vesicles
FOR	Functional overreaching
HIIT	High-intensity interval training
ISR	Integrated stress response
mTOR	Mechanistic target of rapamycin
NF-kB	Nuclear factor kappa B
NFOR	Non-functional overreaching
NOX2	NADPH oxidase 2
Nrf2	Nuclear factor erythroid 2-related factor 2
OTS	Overtraining syndrome
RFI	Recovery Failure Index
ROS	Reactive oxygen species
SIRT1	Sirtuin 1
TFEB	Transcription factor EB
TFE3	Transcription factor E3
UPRmt	Mitochondrial unfolded protein response
VO2max	Maximal oxygen uptake

## References

[1] Meeusen R., Duclos M., Foster C., Fry A., Gleeson M., Nieman D., Raglin J., Rietjens G., Steinacker J., Urhausen A. (2013). Prevention, diagnosis, and treatment of the overtraining syndrome: Joint consensus statement of the European College of Sport Science and the American College of Sports Medicine. Med. Sci. Sports Exerc..

[2] Kreher J.B., Schwartz J.B. (2012). Overtraining syndrome: A practical guide. Sports Health A Multidiscip. Approach.

[3] Cadegiani F.A., da Silva P.H.L., Abrao T.C.P., Kater C.E. (2020). Diagnosis of overtraining syndrome: Results of the Endocrine and Metabolic Responses on Overtraining Syndrome study: EROS-DIAGNOSIS. J. Sports Med..

[4] Carrard J., Rigort A.-C., Appenzeller-Herzog C., Colledge F., Konigstein K., Hinrichs T., Schmidt-Trucksass A. (2022). Diagnosing overtraining syndrome: A scoping review. Sports Health.

[5] Armstrong L.E., Bergeron M.F., Lee E.C., Mershon J.E., Armstrong E.M. (2022). Overtraining syndrome as a complex systems phenomenon. Front. Netw. Physiol..

[6] Buchwald R.L., Buchwald J., Lehtonen E., Peltonen J.E., Uusitalo A.L.T. (2025). A comprehensive analysis of overtraining syndrome in athletes and recreational exercisers. Int. J. Sports Med..

[7] Nieman D.C., Groen A.J., Pugachev A., Vacca G. (2018). Detection of functional overreaching in endurance athletes using proteomics. Proteomes.

[8] Merritt E.K., Nieman D.C., Toone B.R., Groen A., Pugachev A. (2019). Proteomic markers of non-functional overreaching during the Race Across America (RAAM): A case study. Front. Physiol..

[9] Cadegiani F.A., Kater C.E. (2019). Novel insights of overtraining syndrome discovered from the EROS study. BMJ Open Sport Exerc. Med..

[10] Cadegiani F.A., Kater C.E. (2019). Basal hormones and biochemical markers as predictors of overtraining syndrome in male athletes: The EROS-BASAL study. J. Athl. Train..

[11] Cheng A.J., Jude B., Lanner J.T. (2020). Intramuscular mechanisms of overtraining. Redox Biol..

[12] Roussel O.P., Pignanelli C., Hubbard E.F., Coates A.M., Cheng A.J., Burr J.F., Power G.A. (2024). Effects of intensified training with insufficient recovery on joint level and single muscle fibre mechanical function: The role of myofibrillar Ca^2+^ sensitivity. Appl. Physiol. Nutr. Metab..

[13] Watanabe D., Wada M. (2025). Cellular mechanisms underlying overreaching in skeletal muscle following excessive high-intensity interval training. Am. J. Physiol. Cell Physiol..

[14] Sanfrancesco V.C., Hood D.A. (2025). Acute contractile activity induces the activation of the mitochondrial integrated stress response and the transcription factor ATF4. J. Appl. Physiol..

[15] Fiala O., Hanzlova M., Borska L., Fiala Z., Holmannova D. (2025). Beyond physical exhaustion: Understanding overtraining syndrome through the lens of molecular mechanisms and clinical manifestation. Sports Med. Health Sci..

[16] Haller N., Behringer M., Reichel T., Wahl P., Simon P., Kruger K., Zimmer P., Stoggl T. (2023). Blood-based biomarkers for managing workload in athletes: Considerations and recommendations for evidence-based use of established biomarkers. Sports Med..

[17] Bellinger P. (2020). Functional overreaching in endurance athletes: A necessity or cause for concern?. Sports Med..

[18] Sellami M., Elrayess M.A., Puce L., Bragazzi N.L. (2022). Molecular big data in sports sciences: State-of-art and future prospects of OMICS-based sports sciences. Front. Mol. Biosci..

[19] Muniz-Santos R., Magno-França A., Jurisica I., Leite G.S.F., Miarka B. (2023). From microcosm to macrocosm: The -omics, multiomics, and sportomics approaches in exercise and sports. OMICS: A J. Integr. Biol..

[20] Furrer R., Hawley J.A., Handschin C. (2023). The molecular athlete: Exercise physiology from mechanisms to medals. Physiol. Rev..

[21] Cervone D.T., Moreno-Justicia R., Viatte C., Helge J.W., Larsen S., Barrès R. (2024). Mass spectrometry-based proteomics approaches to interrogate skeletal muscle adaptations to exercise. Scand. J. Med. Sci. Sports.

[22] Mănescu D.C. (2026). Training Load Oscillation and Epigenetic Plasticity: Molecular Pathways Connecting Energy Metabolism and Athletic Personality. Int. J. Mol. Sci..

[23] Roete A.J., Elferink-Gemser M.T., Otter R.T.A., Stoter I.K., Lamberts R.P. (2021). A systematic review on markers of functional overreaching in endurance athletes. Int. J. Sports Physiol. Perform..

[24] Chow L.S., Gerszten R.E., Taylor J.M., Pedersen B.K., van Praag H., Trappe S., Febbraio M.A., Galis Z.S., Gao Y., Haus J.M. (2022). Exerkines in health, resilience and disease. Nat. Rev. Endocrinol..

[25] Dvořáková K., Paludo A.C., Wagner H., Puda R., Gimunová M., Kumstát M. (2024). A literature review of biomarkers used for diagnosis of relative energy deficiency in sport. Front. Sports Act. Living.

[26] Suzuki D., Suzuki Y. (2024). Identifying and analyzing low energy availability in athletes: The role of biomarkers and red blood cell turnover. Nutrients.

[27] Mănescu A.M., Tudor A.C., Dinciu C.C., Hangu S.Ș., Mărgărit I.R., Tudor V., Mănescu C.O., Ciomag R.V., Rădulescu M.L., Hangu C. (2025). Interpretable Machine Learning on Simulation-Derived Biomechanical Features for Hamstrings-Quadriceps Imbalance Detection in Running. Sports.

[28] Cadegiani F.A., Kater C.E. (2017). The hypothalamic-pituitary-adrenal axis in overtraining syndrome. Sports Med. Open.

[29] Cadegiani F.A., Kater C.E. (2017). Hormonal aspects of overtraining syndrome: A systematic review. BMC Sports Sci. Med. Rehabil..

[30] Anderson T., Wideman L., Cadegiani F.A., Kater C.E. (2021). Effects of overtraining status on the cortisol awakening response—Endocrine and metabolic responses on overtraining syndrome (EROS-CAR). Int. J. Sports Physiol. Perform..

[31] Cadegiani F.A., Kater C.E. (2018). Hormonal response to a non-exercise stress test in athletes with overtraining syndrome: Results from the Endocrine and Metabolic Responses on Overtraining Syndrome (EROS)—EROS-STRESS. J. Sci. Med. Sport.

[32] Cadegiani F.A., Kater C.E. (2019). Novel causes and consequences of overtraining syndrome: The EROS-DISRUPTORS study. BMC Sports Sci. Med. Rehabil..

[33] Stoian M., Mănescu D.C. (2026). Training-Fuel Coupling (TFC): A Molecular Sports Nutrition Framework for Energy Availability, Chrono-Nutrition, and Performance Optimization. Nutrients.

[34] Hargreaves M., Spriet L.L. (2020). Skeletal muscle energy metabolism during exercise. Nat. Metab..

[35] Coates A.M., Thompson K.M.A., Grigore M.M., Baker R.E., Pignanelli C., Robertson A.A., Frangos S.M., Cheung C.P., Burr J.F. (2024). Altered carbohydrate oxidation during exercise in overreached endurance athletes is applicable to training monitoring with continuous glucose monitors. Scand. J. Med. Sci. Sports.

[36] Ackerman K.E., Holtzman B., Cooper K.M., Flynn E.F., Bruinvels G., Tenforde A.S., Popp K.L., Simpkin A.J., Parziale A.L. (2019). Low energy availability surrogates correlate with health and performance consequences of relative energy deficiency in sport. Br. J. Sports Med..

[37] Keay N., Francis G., Entwistle I., Hind K. (2019). Clinical evaluation of education relating to nutrition and skeletal loading in competitive male road cyclists at risk of relative energy deficiency in sports (RED-S): 6-month randomised controlled trial. BMJ Open Sport Exerc. Med..

[38] Civil R., Lamb A., Loosmore D., Ross L., Livingstone K., Strachan F., Galloway S.D.R., Close G.L., Sale C. (2019). Assessment of dietary intake, energy status, and factors associated with RED-S in vocational female ballet students. Front. Nutr..

[39] Condo D., Lohman R., Kelly M., Carr A. (2019). Nutritional intake, sports nutrition knowledge and energy availability in female Australian rules football players. Nutrients.

[40] Fudge B.W., Westerterp K.R., Kiplamai F.K., Onywera V.O., Boit M.K., Kayser B., Pitsiladis Y.P. (2006). Evidence of negative energy balance using doubly labelled water in elite Kenyan endurance runners prior to competition. Br. J. Nutr..

[41] Tokuyama M., Seino J., Sakuraba K., Suzuki Y. (2021). Possible association of energy availability with transferrin saturation and serum iron during summer camp in male collegiate rugby players. Nutrients.

[42] Areta J.L., Hopkins W.G. (2018). Skeletal muscle glycogen content at rest and during endurance exercise in humans: A meta-analysis. Sports Med..

[43] Ishibashi A., Kojima C., Tanabe Y., Yamada T., Miyakawa T., Iwayama K., Kamei A., Takahashi H., Nabekura Y. (2020). Effect of low energy availability during three consecutive days of endurance training on iron metabolism in male long distance runners. Physiol. Rep..

[44] Kojima C., Ishibashi A., Tanabe Y., Yamada T., Ebi K., Kamei A., Takahashi H., Maruyama T., Nabekura Y. (2020). Muscle glycogen content during endurance training under low energy availability. Med. Sci. Sports Exerc..

[45] Olsson K.-E., Saltin B. (1970). Variation in total body water with muscle glycogen changes in man. Acta Physiol. Scand..

[46] Sherman W.M., Plyley M.J., Sharp R.L., Wright D.A., Simonsen J.C., Shephard R.J. (1982). Muscle glycogen storage and its relationship with water. Int. J. Sports Med..

[47] Brown M.A., Howatson G., Quin E., Redding E., Stevenson E.J. (2017). Energy intake and energy expenditure of pre-professional female contemporary dancers. PLoS ONE.

[48] Mănescu A.M., Hangu S.Ș., Mănescu D.C. (2025). Nutritional Supplements for Muscle Hypertrophy: Mechanisms and Morphology-Focused Evidence. Nutrients.

[49] Kent-Braun J.A., Fitts R.H., Christie A. (2012). Skeletal muscle fatigue. Compr. Physiol..

[50] Enoka R.M., Duchateau J. (2008). Muscle fatigue: What, why and how it influences muscle function. J. Physiol..

[51] Allen D.G., Lamb G.D., Westerblad H. (2008). Skeletal muscle fatigue: Cellular mechanisms. Physiol. Rev..

[52] Gundersen K. (2011). Excitation-transcription coupling in skeletal muscle: The molecular pathways of exercise. Biol. Rev. Camb. Philos. Soc..

[53] Henríquez-Olguín C., Knudsen J.R., Raun S.H., Li Z., Dalbram E., Treebak J.T., Sylow L., Holmdahl R., Jaimovich E., Jensen T.E. (2019). Cytosolic ROS production by NADPH oxidase 2 regulates muscle glucose uptake during exercise. Nat. Commun..

[54] Gallego-Selles A., Martin-Rincon M., Martinez-Canton M., Perez-Valera M., Martin-Rodriguez S., Gelabert-Rebato M., Santana A., Morales-Alamo D., Dorado C., Calbet J.A.L. (2020). Regulation of Nrf2/Keap1 signalling in human skeletal muscle during exercise to exhaustion in normoxia, severe acute hypoxia and post-exercise ischaemia: Influence of metabolite accumulation and oxygenation. Redox Biol..

[55] Yamada M., Iwata M., Warabi E., Oishi H., Lira V.A., Okutsu M. (2019). p62/SQSTM1 and Nrf2 are essential for exercise-mediated enhancement of antioxidant protein expression in oxidative muscle. FASEB J..

[56] Galvan-Alvarez V., Gallego-Selles A., Martinez-Canton M., Garcia-Gonzalez E., Gelabert-Rebato M., Ponce-Gonzalez J.G., Larsen S., Morales-Alamo D., Losa-Reyna J., Perez-Suarez I. (2023). Antioxidant enzymes and Nrf2/Keap1 in human skeletal muscle: Influence of age, sex, adiposity and aerobic fitness. Free Radic. Biol. Med..

[57] Powers S.K., Radak Z., Ji L.L., Jackson M.J. (2024). Reactive oxygen species promote endurance exercise-induced adaptations in skeletal muscles. J. Sport Health Sci..

[58] Egan B., Zierath J.R. (2013). Exercise metabolism and the molecular regulation of skeletal muscle adaptation. Cell Metab..

[59] Camera D.M., Smiles W.J., Hawley J.A. (2016). Exercise-induced skeletal muscle signaling pathways and human athletic performance. Free Radic. Biol. Med..

[60] Denham J., Marques F.Z., O’Brien B.J., Charchar F.J. (2014). Exercise: Putting action into our epigenome. Sports Med..

[61] Langfort J., Viese M., Ploug T., Dela F. (2003). Time course of GLUT4 and AMPK protein expression in human skeletal muscle during one month of physical training. Scand. J. Med. Sci. Sports.

[62] Röckl K.S., Witczak C.A., Goodyear L.J. (2008). Signaling mechanisms in skeletal muscle: Acute responses and chronic adaptations to exercise. IUBMB Life.

[63] Gehlert S., Weinisch P., Römisch-Margl W., Maier A., Weise M., Kienberger H., Hoene M., Geiger P.C. (2022). Effects of acute and chronic resistance exercise on the skeletal muscle metabolome. Metabolites.

[64] Schranner D., Kastenmüller G., Schönfelder M., Römisch-Margl W., Wackerhage H. (2020). Metabolite concentration changes in humans after a bout of exercise: A systematic review of exercise metabolomics studies. Sports Med. Open.

[65] Mănescu D.C., Plăstoi C.D., Petre R.L., Mărgărit I.R., Mănescu A.M., Pîrvan A. (2026). Metabolic Overdrive in Elite Sport: A Systems Model of AMPK-mTOR Oscillation, NAD+ Economy, and Epigenetic Drift. Int. J. Mol. Sci..

[66] Moradi N., Sanfrancesco V.C., Champsi S., Hood D.A. (2024). Regulation of lysosomes in skeletal muscle during exercise, disuse and aging. Free Radic. Biol. Med..

[67] Boardman N.T., Trani G., Scalabrin M., Romanello V., Wust R.C.I. (2023). Intracellular to interorgan mitochondrial communication in striated muscle in health and disease. Endocr. Rev..

[68] Rocchi A., He C. (2017). Regulation of exercise-induced autophagy in skeletal muscle. Curr. Pathobiol. Rep..

[69] Vainshtein A., Hood D.A. (2016). The regulation of autophagy during exercise in skeletal muscle. J. Appl. Physiol..

[70] Gaspar R.S., Katashima C.K., Leite N.C., Silva V.R.R., Lenhare L., Crisol B.M., Cordeiro A.V., Moura L.P., Ropelle E.R., Pauli J.R. (2023). Physical exercise elicits UPRmt in the skeletal muscle: The role of c-Jun N-terminal kinase. Mol. Metab..

[71] Guan Y., Drake J.C., Yan Z. (2019). Exercise-induced mitophagy in skeletal muscle and heart. Exerc. Sport Sci. Rev..

[72] Chatzinikita E., Maridaki M., Palikaras K., Koutsilieris M., Philippou A. (2023). The role of mitophagy in skeletal muscle damage and regeneration. Cells.

[73] Diaz-Castro F., Castro-Sepulveda M., Rivera P., Botella J., Cancino J., Figueroa A.M., Gutiérrez J., Cantin C., Deldicque L., Zbinden-Foncea H. (2024). A single bout of resistance exercise triggers mitophagy, potentially involving the ejection of mitochondria in human skeletal muscle. Acta Physiol..

[74] Drake J.C., Wilson R.J., Yan Z. (2016). Molecular mechanisms for mitochondrial adaptation to exercise training in skeletal muscle. FASEB J..

[75] Gan Z., Fu T., Kelly D.P., Vega R.B. (2018). Skeletal muscle mitochondrial remodeling in exercise and diseases. Cell Res..

[76] Casuso R.A., Huertas J.R. (2020). The emerging role of skeletal muscle mitochondrial dynamics in exercise and ageing. Ageing Res. Rev..

[77] Wang Y., Li J., Zhang Z., Wang R., Bo H., Zhang Y. (2023). Exercise improves the coordination of the mitochondrial unfolded protein response and mitophagy in aging skeletal muscle. Life.

[78] Apablaza P., Llanos P., Figueroa R., Velásquez K., Cruz R., Fernandez-Verdejo R., Castro-Sepulveda M., Jaimovich E. (2023). Exercise induces an augmented skeletal muscle mitochondrial unfolded protein response in a mouse model of obesity produced by a high-fat diet. Int. J. Mol. Sci..

[79] Cordeiro A.V., Peruca G.F., Braga R.R., Brícola R.S., Lenhare L., Silva V.R.R., Anaruma C.P., Katashima C.K., Crisol B.M., Barbosa L.T. (2021). High-intensity exercise training induces mitonuclear imbalance and activates the mitochondrial unfolded protein response in the skeletal muscle of aged mice. GeroScience.

[80] Kim K., Kim Y.-H., Lee S.-H., Jeon M.-J., Park S.-Y., Doh K.-O., Kim H.-S., Kim T.-Y. (2014). Effect of exercise intensity on unfolded protein response in skeletal muscle of rat. Korean J. Physiol. Pharmacol..

[81] Richards B.J., Slavin M.B., Oliveira A.N., Gurtler A., Vainshtein A., Hood D.A. (2023). Mitochondrial protein import and UPRmt in skeletal muscle remodeling and adaptation. Semin. Cell Dev. Biol..

[82] Mishra P., Varuzhanyan G., Pham A.H., Chan D.C. (2015). Mitochondrial dynamics is a distinguishing feature of skeletal muscle fiber types and regulates organellar compartmentalization. Cell Metab..

[83] Sebastián D., Hernández-Alvarez M.I., Segalés J., Sorianello E., Muñoz J.P., Sala D., Waget A., Liesa M., Paz J.C., Gopalacharyulu P. (2016). Mfn2 deficiency links age-related sarcopenia and impaired autophagy to activation of an adaptive mitophagy pathway. EMBO J..

[84] de Brito O.M., Scorrano L. (2008). Mitofusin 2 tethers endoplasmic reticulum to mitochondria. Nature.

[85] Filadi R., Greotti E., Turacchio G., Luini A., Pozzan T., Pizzo P. (2015). Mitofusin 2 ablation increases endoplasmic reticulum–mitochondria coupling. Proc. Natl. Acad. Sci. USA.

[86] Naon D., Zaninello M., Giacomello M., Varanita T., Grespi F., Lakshminaranayan S., Serafini A., Semenzato M., Herkenne S., Hernández-Alvarez M.I. (2016). Critical reappraisal confirms that Mitofusin 2 is an endoplasmic reticulum–mitochondria tether. Proc. Natl. Acad. Sci. USA.

[87] Polakovičová M., Musil P., Laczo E., Hamar D., Kyselovič J. (2016). Circulating microRNAs as potential biomarkers of exercise response. Int. J. Mol. Sci..

[88] Xu T., Liu Q., Yao J., Dai Y., Wang H., Xiao J. (2015). Circulating microRNAs in response to exercise. Scand. J. Med. Sci. Sports.

[89] Sapp R.M., Shill D.D., Roth S.M., Hagberg J.M. (2017). Circulating microRNAs in acute and chronic exercise: More than mere biomarkers. J. Appl. Physiol..

[90] Fernández-Sanjurjo M., de Gonzalo-Calvo D., Fernández-García B., Díez-Robles S., Martínez-Canal Á., Olmedillas H., Dávalos A., Iglesias-Gutiérrez E. (2018). Circulating microRNA as emerging biomarkers of exercise. Exerc. Sport Sci. Rev..

[91] Vechetti I.J., Valentino T., Mobley C.B., McCarthy J.J. (2021). The role of extracellular vesicles in skeletal muscle and systematic adaptation to exercise. J. Physiol..

[92] Nederveen J.P., Warnier G., Di Carlo A., Nilsson M.I., Tarnopolsky M.A. (2021). Extracellular vesicles and exosomes: Insights from exercise science. Front. Physiol..

[93] Darragh I.A.J., O’Driscoll L., Egan B. (2021). Exercise training and circulating small extracellular vesicles: Appraisal of methodological approaches and current knowledge. Front. Physiol..

[94] Lombardo M., Aiello G., Fratantonio D., Karav S., Baldelli S. (2024). Functional role of extracellular vesicles in skeletal muscle physiology and sarcopenia: The importance of physical exercise and nutrition. Nutrients.

[95] Park S., Moon H.Y. (2022). Urinary extracellular vesicle as a potential biomarker of exercise-induced fatigue in young adult males. Eur. J. Appl. Physiol..

[96] Conkright W.R., Kargl C.K., Hubal M.J., Tiede D.R., Beckner M.E., Sterczala A.J., Krajewski K.T., Martin B.J., Flanagan S.D., Greeves J.P. (2024). Acute resistance exercise modifies extracellular vesicle microRNAs targeting anabolic gene pathways: A prospective cohort study. Med. Sci. Sports Exerc..

[97] Jakicic J.M., Kraus W.E., Bhasin S., Buford T.W., Evans M., Haskell W.L., Poirier P., Sacks F.M., White J.P., MoTrPAC Study Group (2024). Molecular Transducers of Physical Activity Consortium (MoTrPAC): Human studies design and protocol. J. Appl. Physiol..

[98] Johnson C.H., Ivanisevic J., Siuzdak G. (2016). Metabolomics: Beyond biomarkers and towards mechanisms. Nat. Rev. Mol. Cell Biol..

[99] Voisin S., Almen M.S., Moslehi N., Lindholm M.E., Ekblom Ö., Ekblom B., Kulyté A., Jansson E., Sundberg C.J. (2024). Exercise is associated with younger methylome and transcriptome profiles in human skeletal muscle. Aging Cell.

[100] Jacques M., Landen S., Sharples A.P., Garnham A., Pillon N.J., Lindholm M.E., Hawley J.A., Zierath J.R., Voisin S. (2025). Molecular landscape of sex- and modality-specific exercise adaptation in human skeletal muscle through large-scale multi-omics integration. Cell Rep..

[101] Amar D., Lindholm M.E., Norrbom J., Wheeler M.T., Rivas M.A., Ashley E.A. (2021). Time trajectories in the transcriptomic response to exercise-a meta-analysis. Nat. Commun..

[102] MoTrPAC Study Group (2024). Temporal dynamics of the multi-omic response to endurance exercise training. Nature.

[103] Sanford J.A., Nogiec C.D., Lindholm M.E., Adkins J.N., Amar D., Dasari S., Drugan J.K., Fernandez F.M., Radom-Aizik S., Schenk S. (2020). Molecular Transducers of Physical Activity Consortium (MoTrPAC): Mapping the Dynamic Responses to Exercise. Cell.

[104] Pillon N.J., Gabriel B.M., Dollet L., Smith J.A.B., Sardón Puig L., Botella J., Bishop D.J., Krook A., Zierath J.R. (2020). Transcriptomic profiling of skeletal muscle adaptations to exercise and inactivity. Nat. Commun..

[105] Makhnovskii P.A., Bokov R.O., Kolpakov F.A., Popov D.V. (2021). Transcriptomic signatures and upstream regulation in human skeletal muscle adapted to disuse and aerobic exercise. Int. J. Mol. Sci..

[106] Maasar M.-F., Turner D.C., Gorski P.P., Seaborne R.A., Strauss J.A., Shepherd S.O., Cocks M., Pillon N.J., Zierath J.R., Hulton A.T. (2021). The comparative methylome and transcriptome after change of direction compared to straight line running exercise in human skeletal muscle. Front. Physiol..

[107] Zhao J., Wang Y., Zhao D., Li B., Li M., Zhang S., Sun D., Zhang H., Wang T., Wang J. (2020). Integration of metabolomics and proteomics to reveal metabolic characteristics of high intensity interval training. Analyst.

[108] Pechlivanis A., Papaioannou K.G., Tsalis G., Saraslanidis P., Mougios V., Theodoridis G.A. (2015). Monitoring the response of the human urinary metabolome to brief maximal exercise by a combination of RP-UPLC-MS and 1H NMR spectroscopy. J. Proteome Res..

[109] Pellegrino J.K., Anthony T.G., Gillies P.J., Arent S.M. (2022). The exercise metabolome: Acute aerobic and anaerobic signatures. J. Int. Soc. Sports Nutr..

[110] Khoramipour K., Sandbakk Ø., Keshteli A.H., Gaeini A.A., Wishart D.S., Chamari K. (2022). Metabolomics in exercise and sports: A systematic review. Sports Med..

[111] Mănescu D.C., Tudor A., Mănescu A.M., Mărgărit I.R., Mănescu C.O., Prisăcaru C., Păun L., Tudor V. (2026). Antioxidants and Exercise: A Redox-Informed Framework for Training Adaptation, Performance, and Recovery. Antioxidants.

[112] Morville T., Sahl R.E., Moritz T., Helge J.W., Clemmensen C. (2020). Plasma metabolome profiling of resistance exercise and endurance exercise in humans. Cell Rep..

[113] Lewis N.A., Collins D., Pedlar C.R., Rogers J.P. (2015). Can clinicians and scientists explain and prevent unexplained underperformance syndrome in elite athletes: An interdisciplinary perspective and 2016 update. BMJ Open Sport Exerc. Med..

[114] Urhausen A., Kindermann W. (2002). Diagnosis of overtraining: What tools do we have?. Sports Med..

[115] Carfagno D.G., Hendrix J.C. (2014). Overtraining syndrome in the athlete: Current clinical practice. Curr. Sports Med. Rep..

